# *N*′-[(*E*)-(3-Fluoro­pyridin-2-yl)methyl­idene]benzohydrazide monohydrate

**DOI:** 10.1107/S1600536812035179

**Published:** 2012-08-15

**Authors:** Yamuna Nair, M. Sithambaresan, M. R. Prathapachandra Kurup

**Affiliations:** a Department of Chemical Oceanography, Cochin University of Science and Technology, Lakeside Campus, Kochi 682 016, India; b Department of Chemistry, Faculty of Science, Eastern University, Sri Lanka, Chenkalady, Sri Lanka; c Department of Applied Chemistry, Cochin University of Science and Technology, Kochi 682 022, India

## Abstract

The title compound, C_13_H_10_FN_3_O·H_2_O, exists in the *E* conformation with respect to the azomethane C=N double bond. The mol­ecule is close to planar with a maximum deviation of 0.286 (2) Å. The pyridine ring is essentially coplanar with the central C(= O)N_2_C unit [dihedral angle = 2.02 (3)°] and the phenyl ring exhibits a dihedral angle of 14.41 (10)° with respect to the central unit. The crystal structure features O—H⋯N, N—H⋯O and O—H⋯O hydrogen-bond inter­actions between the solvent water and the benzohydrazide mol­ecules, as well as C—H⋯O hydrogen bonds and C—F⋯π [3.0833 (18) Å] inter­actions.

## Related literature

For background to the use of benzohydrazides as catalysts, see: Heravi *et al.* (2007 ▶); Hou *et al.* (2005 ▶) and for their biological activity, see: Sreeja *et al.* (2004 ▶). For the synthesis of related compounds, see: Fun *et al.* (2008 ▶). For related structures, see Mangalam *et al.* (2009 ▶).
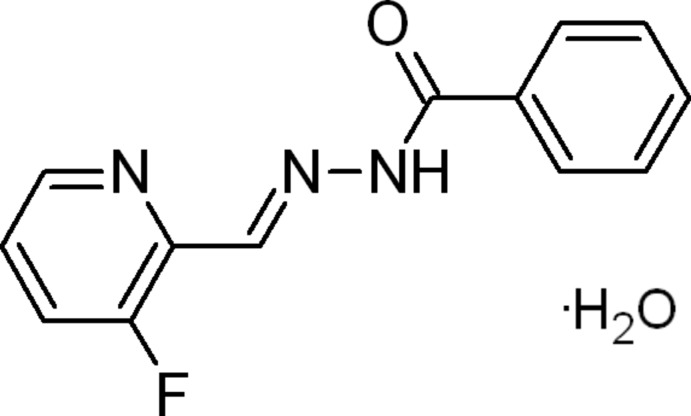


## Experimental

### 

#### Crystal data

C_13_H_10_FN_3_O·H_2_O
*M* *_r_* = 261.26
Orthorhombic, 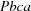

*a* = 8.2540 (4) Å
*b* = 11.5489 (4) Å
*c* = 26.1962 (11) Å
*V* = 2497.14 (18) Å^3^
*Z* = 8
Mo *K*α radiation
μ = 0.11 mm^−1^
*T* = 296 K
0.35 × 0.30 × 0.25 mm

#### Data collection

Bruker Kappa APEXII CCD diffractometer
Absorption correction: multi-scan (*SADABS*; Bruker, 2004 ▶) *T*_min_ = 0.964, *T*_max_ = 0.974
34264 measured reflections
2192 independent reflections
1817 reflections with *I* > 2σ(*I*)
*R*_int_ = 0.027

#### Refinement

*R*[*F* ^2^ > 2σ(*F* ^2^)] = 0.032
*wR*(*F* ^2^) = 0.096
*S* = 1.07
2190 reflections
185 parameters
H atoms treated by a mixture of independent and constrained refinement
Δρ_max_ = 0.18 e Å^−3^
Δρ_min_ = −0.13 e Å^−3^

### 

Data collection: *APEX2* (Bruker, 2004 ▶); cell refinement: *APEX2* and *SAINT* (Bruker, 2004 ▶); data reduction: *SAINT* and *XPREP* (Bruker, 2004 ▶); program(s) used to solve structure: *SHELXS97* (Sheldrick, 2008 ▶); program(s) used to refine structure: *SHELXL97* (Sheldrick, 2008 ▶); molecular graphics: *ORTEP-3* (Farrugia, 1997 ▶) and *DIAMOND* (Brandenburg, 2010 ▶); software used to prepare material for publication: *SHELXL97* and *publCIF* (Westrip, 2010 ▶).

## Supplementary Material

Crystal structure: contains datablock(s) global, I. DOI: 10.1107/S1600536812035179/zl2494sup1.cif

Structure factors: contains datablock(s) I. DOI: 10.1107/S1600536812035179/zl2494Isup2.hkl

Supplementary material file. DOI: 10.1107/S1600536812035179/zl2494Isup3.cml

Additional supplementary materials:  crystallographic information; 3D view; checkCIF report

## Figures and Tables

**Table 1:** Hydrogen-bond geometry (Å, °)

*D*—H⋯*A*	*D*—H	H⋯*A*	*D*⋯*A*	*D*—H⋯*A*
N3—H3′⋯O1*W*	0.901 (18)	1.914 (18)	2.7917 (17)	164.3 (16)
O1*W*—H1*A*⋯O1^i^	0.84 (3)	2.08 (3)	2.9187 (19)	172 (2)
O1*W*—H1*A*⋯N2^i^	0.84 (3)	2.48 (2)	2.9494 (17)	116.1 (19)
O1*W*—H1*B*⋯N1^i^	0.89 (2)	1.95 (3)	2.8420 (18)	178 (2)
C9—H9⋯O1*W*	0.93	2.30	3.209 (2)	165

Symmetry code: (i) 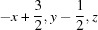.

**Fig. 1.**
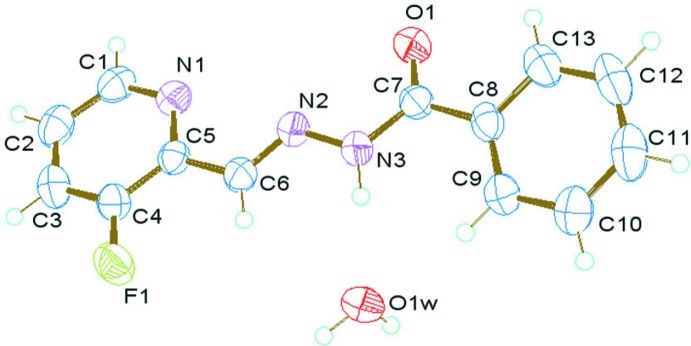
ORTEP diagram of N'-[(E)-(3-fluoropyridin-2-yl)methylidene]benzohydrazide monohydrate with 50% probability ellipsoids.

**Fig. 2.**
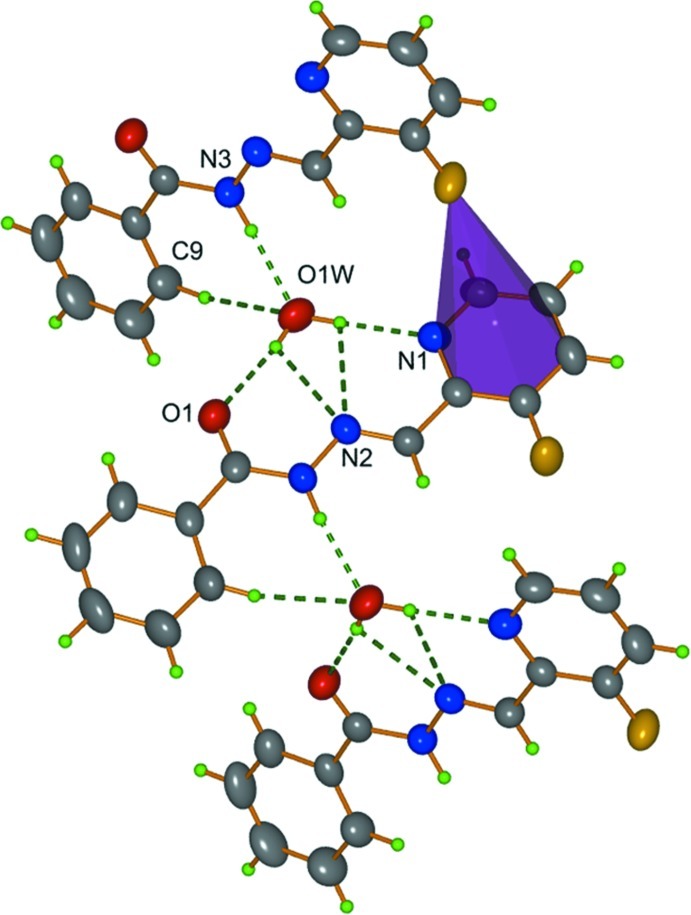
Hydrogen-bonding interactions showing an infinite chain in the crystal structure of N'-[(E)-(3-fluoropyridin-2-yl)methylidene]benzohydrazide hydrate and the interconnection of the chains via weak C–F···π interactions.

## supplementary crystallographic information

### Comment

Interest in coordination chemistry of benzohydrazide has been a subject of enthusiastic research since their complexes show a wide range of catalytic properties (Heravi *et al.*, 2007; Hou *et al.*, 2005). Benzohydrazone derivatives are also important due to their wide spectrum of biological activities (Sreeja *et al.*, 2004).

This molecule adopts an *E* conformation with respect to the C6=N2 bond and it exists in the amido form with a C7=O1 bond length of 1.2258 (17) Å which is very close to the reported C=O bond length of a similar structure (Mangalam *et al.*, 2009). The O1 and N2 atoms are in a *Z* conformation with respect to C7–N3 having a torsional angle of -0.4 (2)°. The molecule is almost planar with a maximum deviation of 0.286 (2) Å for the atom C12 from its least square plane. The pyridyl ring is essentially coplanar with the central C(=O)N_2_C unit (dihedral angle 2.02 (3) °), the phenyl ring exhibits a dihedral angle of 14.41 (10)° with respect to the central unit.

The water molecule forms six H-bonds with two different benzohydrazone molecules. Hydrogen bond interactions such as O–H···N, O–H···O, N–H···O and C–H···O are present in the crystal system between the H atoms attached to the O1W atom and N1, N2, N3, C9 and O1 atoms of two adjacent molecules with D···A distances of 2.842 (2), 2.9495 (17), 2.7917 (17), 3.209 (2) and 2.9188 (18) Å respectively as shown in Table 1. Both H-atoms of the water molecule form bifurcated hydrogen bonds with the azomethine nitrogen, the pyridyl nitrogen and the carbonyl oxygen atoms of one neighboring molecule (Fig. 2). The water molecule acts as a hydrogen bond acceptor towards another benzohydrazone molecule through an N–H···O hydrogen bond. Through these interactions the molecules are interconnected through the water molecule to form infinite chains parallel to the *b* axis of the unit cell (Fig. 2). Benzohydrazone molecules within these chains also interact through weak C–F···π [3.0833 (18) Å] interactions (Fig. 2) that augment the stronger O–H···N, O–H···O, N–H···O hydrogen bonds.

### Experimental

The title compound was prepared by adapting a reported procedure (Fun *et al.*, 2008). A solution of 3-fluoropyridine-2-carbaldehyde (1.25 g,1 mmol) in ethanol (10 ml) was mixed with an ethanolic solution (10 ml) of benzohydrazide (1.36 g,1 mmol). The mixture was boiled under reflux for 12 h after adding few drops of glacial acetic acid and then cooled to room temperature. Colorless block shaped crystals, suitable for single-crystal analysis, were obtained in 61.8% yield after slow evaporation of the solution in air for a few days. ^1^H NMR spectrum, DMSO-d_6_, δ, p.p.m.: 12.12 (s, 1H, NH), 8.66 (s, 1H, CH=N), 8.53 (d, 1H, py–H(C1)), 7.54–7.95 (m, 7H, Ar–H (C2, C3, C9, C10, C11, C12, C13)). IR spectrum, ν (cm^-1^): 3421, 3059, 1683,1650, 1597, 1441, 1350, 1295, 1273, 1167, 1134, 1073, 922, 801, 706, 674.

### Refinement

The atoms H3', H1A and H1B were located from a difference Fourier map and refined isotropically. The remaining hydrogen atoms were placed in geometrically idealized positions and constrained to ride on their parent atoms, with C–H distances of 0.93 Å, and with isotropic displacement parameters 1.2 times that of the parent carbon atoms. Omitted owing to bad disagreement were the reflections (0 0 2) and (1 0 2).

### Crystal data

**Table:**

C_13_H_10_FN_3_O·H_2_O	*F*(000) = 1088
*M**_r_* = 261.26	*D*_x_ = 1.390 Mg m^−^^3^
Orthorhombic, *P**b**c**a*	Mo *K*α radiation, λ = 0.71073 Å
Hall symbol: -P 2ac 2ab	Cell parameters from 9884 reflections
*a* = 8.2540 (4) Å	θ = 5.8–54.5°
*b* = 11.5489 (4) Å	µ = 0.11 mm^−^^1^
*c* = 26.1962 (11) Å	*T* = 296 K
*V* = 2497.14 (18) Å^3^	Block, colorless
*Z* = 8	0.35 × 0.30 × 0.25 mm

### Data collection

**Table:**

Bruker Kappa APEXII CCD diffractometer	2192 independent reflections
Radiation source: fine-focus sealed tube	1817 reflections with *I* > 2σ(*I*)
Graphite monochromator	*R*_int_ = 0.027
ω and φ scan	θ_max_ = 25.0°, θ_min_ = 3.1°
Absorption correction: multi-scan (*SADABS*; Bruker, 2004)	*h* = −9→9
*T*_min_ = 0.964, *T*_max_ = 0.974	*k* = −13→13
34264 measured reflections	*l* = −31→31

### Refinement

**Table:**

Refinement on *F*^2^	Secondary atom site location: difference Fourier map
Least-squares matrix: full	Hydrogen site location: inferred from neighbouring sites
*R*[*F*^2^ > 2σ(*F*^2^)] = 0.032	H atoms treated by a mixture of independent and constrained refinement
*wR*(*F*^2^) = 0.096	*w* = 1/[σ^2^(*F*_o_^2^) + (0.0425*P*)^2^ + 0.6736*P*] where *P* = (*F*_o_^2^ + 2*F*_c_^2^)/3
*S* = 1.07	(Δ/σ)_max_ = 0.001
2190 reflections	Δρ_max_ = 0.18 e Å^−^^3^
185 parameters	Δρ_min_ = −0.13 e Å^−^^3^
0 restraints	Extinction correction: *SHELXL97* (Sheldrick, 2008), Fc^*^=kFc[1+0.001xFc^2^λ^3^/sin(2θ)]^-1/4^
Primary atom site location: structure-invariant direct methods	Extinction coefficient: 0.0073 (8)

### Special details

**Table:**

Geometry. All e.s.d.'s (except the e.s.d. in the dihedral angle between two l.s. planes) are estimated using the full covariance matrix. The cell e.s.d.'s are taken into account individually in the estimation of e.s.d.'s in distances, angles and torsion angles; correlations between e.s.d.'s in cell parameters are only used when they are defined by crystal symmetry. An approximate (isotropic) treatment of cell e.s.d.'s is used for estimating e.s.d.'s involving l.s. planes.
Refinement. Refinement of *F*^2^ against ALL reflections. The weighted *R*-factor *wR* and goodness of fit *S* are based on *F*^2^, conventional *R*-factors *R* are based on *F*, with *F* set to zero for negative *F*^2^. The threshold expression of *F*^2^ > σ(*F*^2^) is used only for calculating *R*-factors(gt) *etc*. and is not relevant to the choice of reflections for refinement. *R*-factors based on *F*^2^ are statistically about twice as large as those based on *F*, and *R*- factors based on ALL data will be even larger.

### Fractional atomic coordinates and isotropic or equivalent isotropic displacement parameters (Å^2^)

**Table:**

	*x*	*y*	*z*	*U*_iso_*/*U*_eq_	
F1	0.75071 (13)	0.87133 (8)	1.09485 (3)	0.0629 (3)	
O1	0.82638 (15)	1.12491 (9)	0.86590 (4)	0.0561 (3)	
O1W	0.90084 (17)	0.73757 (10)	0.93534 (6)	0.0600 (3)	
N1	0.60294 (15)	1.12483 (10)	1.03176 (5)	0.0462 (3)	
N2	0.79363 (14)	1.02906 (10)	0.95707 (4)	0.0413 (3)	
N3	0.88214 (15)	0.97616 (11)	0.91928 (4)	0.0418 (3)	
C1	0.51707 (19)	1.17020 (13)	1.06961 (6)	0.0514 (4)	
H1	0.4633	1.2397	1.0637	0.062*	
C2	0.5032 (2)	1.12006 (14)	1.11710 (6)	0.0562 (4)	
H2	0.4420	1.1552	1.1425	0.067*	
C3	0.5812 (2)	1.01773 (15)	1.12616 (6)	0.0557 (4)	
H3	0.5742	0.9811	1.1577	0.067*	
C4	0.66988 (18)	0.97122 (13)	1.08709 (5)	0.0444 (4)	
C5	0.68155 (16)	1.02434 (11)	1.04024 (5)	0.0392 (3)	
C6	0.77636 (17)	0.97353 (12)	0.99866 (5)	0.0416 (3)	
H6	0.8237	0.9010	1.0026	0.050*	
C7	0.89290 (17)	1.03168 (12)	0.87370 (5)	0.0416 (3)	
C8	0.99453 (17)	0.97527 (13)	0.83362 (5)	0.0427 (3)	
C9	1.0457 (2)	0.86099 (14)	0.83555 (6)	0.0543 (4)	
H9	1.0176	0.8148	0.8633	0.065*	
C10	1.1385 (2)	0.81533 (17)	0.79643 (6)	0.0664 (5)	
H10	1.1715	0.7384	0.7978	0.080*	
C11	1.1819 (2)	0.88324 (18)	0.75569 (7)	0.0692 (5)	
H11	1.2441	0.8522	0.7294	0.083*	
C12	1.1337 (2)	0.99666 (18)	0.75360 (6)	0.0650 (5)	
H12	1.1645	1.0427	0.7261	0.078*	
C13	1.03959 (19)	1.04278 (15)	0.79213 (6)	0.0529 (4)	
H13	1.0062	1.1196	0.7903	0.064*	
H3'	0.908 (2)	0.9010 (16)	0.9235 (6)	0.057 (5)*	
H1A	0.838 (3)	0.699 (2)	0.9166 (9)	0.102 (9)*	
H1B	0.902 (3)	0.701 (2)	0.9653 (9)	0.100 (8)*	

### Atomic displacement parameters (Å^2^)

**Table:**

	*U*^11^	*U*^22^	*U*^33^	*U*^12^	*U*^13^	*U*^23^
F1	0.0738 (6)	0.0639 (6)	0.0511 (5)	0.0185 (5)	0.0040 (5)	0.0128 (4)
O1	0.0757 (8)	0.0461 (6)	0.0467 (6)	0.0037 (5)	0.0021 (5)	0.0061 (5)
O1W	0.0761 (8)	0.0452 (6)	0.0589 (8)	−0.0080 (6)	0.0070 (7)	0.0081 (6)
N1	0.0496 (7)	0.0422 (7)	0.0468 (7)	0.0015 (5)	0.0020 (6)	−0.0028 (5)
N2	0.0454 (7)	0.0418 (6)	0.0366 (6)	−0.0004 (5)	0.0024 (5)	−0.0031 (5)
N3	0.0500 (7)	0.0406 (7)	0.0348 (6)	0.0017 (5)	0.0046 (5)	−0.0004 (5)
C1	0.0508 (9)	0.0451 (8)	0.0583 (10)	0.0016 (7)	0.0051 (7)	−0.0096 (7)
C2	0.0529 (9)	0.0628 (10)	0.0528 (10)	−0.0014 (8)	0.0107 (7)	−0.0155 (8)
C3	0.0571 (9)	0.0704 (11)	0.0395 (8)	−0.0017 (8)	0.0053 (7)	−0.0008 (7)
C4	0.0447 (8)	0.0492 (8)	0.0395 (8)	0.0006 (7)	−0.0027 (6)	−0.0008 (6)
C5	0.0381 (7)	0.0417 (7)	0.0377 (7)	−0.0032 (6)	−0.0013 (6)	−0.0039 (6)
C6	0.0459 (8)	0.0401 (8)	0.0388 (7)	0.0034 (6)	−0.0001 (6)	−0.0008 (6)
C7	0.0458 (8)	0.0415 (8)	0.0375 (8)	−0.0089 (6)	−0.0036 (6)	−0.0003 (6)
C8	0.0421 (8)	0.0527 (8)	0.0333 (7)	−0.0109 (6)	−0.0020 (6)	−0.0007 (6)
C9	0.0643 (10)	0.0551 (9)	0.0433 (8)	−0.0037 (8)	0.0112 (8)	−0.0003 (7)
C10	0.0736 (12)	0.0708 (11)	0.0550 (10)	0.0039 (9)	0.0161 (9)	−0.0077 (9)
C11	0.0611 (11)	0.1001 (15)	0.0463 (10)	−0.0034 (10)	0.0143 (8)	−0.0098 (10)
C12	0.0576 (10)	0.0987 (14)	0.0386 (9)	−0.0147 (10)	0.0058 (7)	0.0105 (9)
C13	0.0519 (9)	0.0651 (10)	0.0418 (8)	−0.0110 (8)	−0.0024 (7)	0.0068 (7)

### Geometric parameters (Å, º)

**Table:**

F1—C4	1.3481 (17)	C4—C5	1.376 (2)
O1—C7	1.2258 (17)	C5—C6	1.4639 (19)
O1W—H1A	0.84 (3)	C6—H6	0.9300
O1W—H1B	0.89 (2)	C7—C8	1.494 (2)
N1—C1	1.3266 (19)	C8—C9	1.387 (2)
N1—C5	1.3480 (18)	C8—C13	1.388 (2)
N2—C6	1.2721 (18)	C9—C10	1.384 (2)
N2—N3	1.3736 (16)	C9—H9	0.9300
N3—C7	1.3582 (18)	C10—C11	1.372 (3)
N3—H3'	0.901 (18)	C10—H10	0.9300
C1—C2	1.377 (2)	C11—C12	1.370 (3)
C1—H1	0.9300	C11—H11	0.9300
C2—C3	1.366 (2)	C12—C13	1.381 (2)
C2—H2	0.9300	C12—H12	0.9300
C3—C4	1.368 (2)	C13—H13	0.9300
C3—H3	0.9300		
			
H1A—O1W—H1B	105 (2)	C5—C6—H6	120.2
C1—N1—C5	118.31 (13)	O1—C7—N3	122.14 (13)
C6—N2—N3	116.90 (12)	O1—C7—C8	121.16 (13)
C7—N3—N2	117.28 (12)	N3—C7—C8	116.67 (13)
C7—N3—H3'	123.2 (11)	C9—C8—C13	118.79 (14)
N2—N3—H3'	117.8 (11)	C9—C8—C7	124.09 (13)
N1—C1—C2	123.60 (15)	C13—C8—C7	117.12 (14)
N1—C1—H1	118.2	C10—C9—C8	120.27 (15)
C2—C1—H1	118.2	C10—C9—H9	119.9
C3—C2—C1	118.79 (14)	C8—C9—H9	119.9
C3—C2—H2	120.6	C11—C10—C9	120.18 (18)
C1—C2—H2	120.6	C11—C10—H10	119.9
C2—C3—C4	117.49 (15)	C9—C10—H10	119.9
C2—C3—H3	121.3	C12—C11—C10	120.13 (17)
C4—C3—H3	121.3	C12—C11—H11	119.9
F1—C4—C3	119.21 (13)	C10—C11—H11	119.9
F1—C4—C5	118.78 (13)	C11—C12—C13	120.19 (16)
C3—C4—C5	122.00 (14)	C11—C12—H12	119.9
N1—C5—C4	119.81 (13)	C13—C12—H12	119.9
N1—C5—C6	118.68 (12)	C12—C13—C8	120.43 (16)
C4—C5—C6	121.51 (13)	C12—C13—H13	119.8
N2—C6—C5	119.67 (12)	C8—C13—H13	119.8
N2—C6—H6	120.2		
			
C6—N2—N3—C7	176.10 (12)	N2—N3—C7—O1	−0.4 (2)
C5—N1—C1—C2	−0.2 (2)	N2—N3—C7—C8	177.81 (11)
N1—C1—C2—C3	−0.2 (2)	O1—C7—C8—C9	−166.31 (14)
C1—C2—C3—C4	0.3 (2)	N3—C7—C8—C9	15.5 (2)
C2—C3—C4—F1	178.90 (14)	O1—C7—C8—C13	13.8 (2)
C2—C3—C4—C5	0.1 (2)	N3—C7—C8—C13	−164.45 (13)
C1—N1—C5—C4	0.6 (2)	C13—C8—C9—C10	−0.7 (2)
C1—N1—C5—C6	179.89 (13)	C7—C8—C9—C10	179.37 (15)
F1—C4—C5—N1	−179.40 (12)	C8—C9—C10—C11	0.7 (3)
C3—C4—C5—N1	−0.5 (2)	C9—C10—C11—C12	0.1 (3)
F1—C4—C5—C6	1.4 (2)	C10—C11—C12—C13	−0.8 (3)
C3—C4—C5—C6	−179.78 (14)	C11—C12—C13—C8	0.8 (3)
N3—N2—C6—C5	−179.18 (11)	C9—C8—C13—C12	0.0 (2)
N1—C5—C6—N2	5.7 (2)	C7—C8—C13—C12	179.90 (14)
C4—C5—C6—N2	−175.10 (14)		

### Hydrogen-bond geometry (Å, º)

**Table:**

*D*—H···*A*	*D*—H	H···*A*	*D*···*A*	*D*—H···*A*
N3—H3′···O1*W*	0.901 (18)	1.914 (18)	2.7917 (17)	164.3 (16)
O1*W*—H1*A*···O1^i^	0.84 (3)	2.08 (3)	2.9187 (19)	172 (2)
O1*W*—H1*A*···N2^i^	0.84 (3)	2.48 (2)	2.9494 (17)	116.1 (19)
O1*W*—H1*B*···N1^i^	0.89 (2)	1.95 (3)	2.8420 (18)	178 (2)
C9—H9···O1*W*	0.93	2.30	3.209 (2)	165

Symmetry code: (i) −*x*+3/2, *y*−1/2, *z*.
